# Neurosupportive Role of Vanillin, a Natural Phenolic Compound, on Rotenone Induced Neurotoxicity in SH-SY5Y Neuroblastoma Cells

**DOI:** 10.1155/2015/626028

**Published:** 2015-11-17

**Authors:** Chinnasamy Dhanalakshmi, Thamilarasan Manivasagam, Jagatheesan Nataraj, Arokiasamy Justin Thenmozhi, Musthafa Mohamed Essa

**Affiliations:** ^1^Department of Biochemistry and Biotechnology, Annamalai University, Annamalainagar, Tamil Nadu 608002, India; ^2^Department of Food Science and Nutrition, CAMS, Sultan Qaboos University, Muscat, Oman; ^3^Ageing and Dementia Research Group, Sultan Qaboos University, Muscat, Oman; ^4^Food and Brain Research Foundation, Chennai, Tamil Nadu 600094, India

## Abstract

Vanillin, a phenolic compound, has been reported to offer neuroprotection against experimental Huntington's disease and global ischemia by virtue of its antioxidant, anti-inflammatory, and antiapoptotic properties. The present study aims to elucidate the underlying neuroprotective mechanism of vanillin in rotenone induced neurotoxicity. Cell viability was assessed by exposing SH-SY5Y cells to various concentrations of rotenone (5–200 nM) for 24 h. The therapeutic effectiveness of vanillin against rotenone was measured by pretreatment of vanillin at various concentrations (5–200 nM) and then incubation with rotenone (100 nM). Using effective dose of vanillin (100 nM), mitochondrial membrane potential, levels of reactive oxygen species (ROS), and expression patterns of apoptotic markers were assessed. Toxicity of rotenone was accompanied by the loss of mitochondrial membrane potential, increased ROS generation, release of cyt-c, and enhanced expressions of proapoptotic and downregulation of antiapoptotic indices via the upregulation of p38 and JNK-MAPK pathway proteins. Our results indicated that the pretreatment of vanillin attenuated rotenone induced mitochondrial dysfunction, oxidative stress, and apoptosis. Thus, vanillin may serve as a potent therapeutic agent in the future by virtue of its multiple pharmacological properties in the treatment of neurodegenerative diseases including PD.

## 1. Introduction

Parkinson's disease (PD) is one of the most common, progressive, and age-related neurodegenerative diseases, that is, raised by the selective loss of dopaminergic (DA) neurons in substantia nigra pars compacta and resulting in striatal dopamine depletion leading to movement disorder [1]. Though the cause of PD is not known, most of the knowledge about PD pathology is gathered from various* in vivo* and* in vitro* models. Rotenone, a naturally occurring plant flavonoid and widely used pesticide, is a specific inhibitor of complex I of the mitochondrial respiratory chain and mimicked the symptoms of PD, both* in vivo* and* in vitro* conditions [2–5]. The SH-SY5Y cell line is considered as the excellent cellular model for PD research because this cell line possesses many characteristics (expression of tyrosine hydroxylase, dopamine-beta-hydroxylase, and dopamine transporter) of DAergic neurons [6]. Epidemiological studies suggest that exposure to environmental agents, such as pesticides, may increase the PD risk [7, 8]. Mitochondrial dysfunction has also been linked to PD.* In vitro* rotenone model of PD authenticated the involvement of both mitochondrial dysfunction and environmental exposures in PD.

The* in vitro* studies indicated that rotenone can easily cross the biological membranes, due to its lipophilic nature, and could access the cytoplasm of dopaminergic neurons easily [3]. Furthermore it could enter into mitochondria and inhibit mitochondrial complex I activity. It can also induce ROS generation and loss of mitochondrial membrane potential (MMP) and release cytochrome c (cyt-c) from mitochondria, which in turn activate the caspase cascades via regulation of JNK, p38, and ERK MAPK pathways [2, 9].

**Figure 1:**
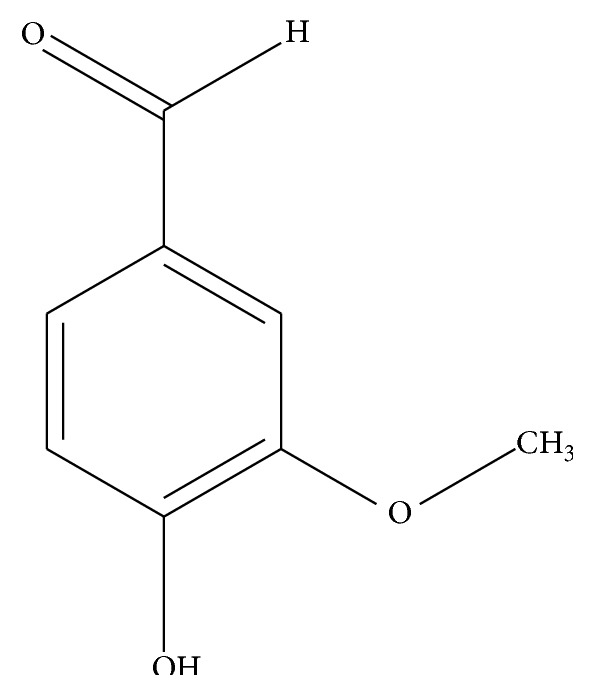
Structure of vanillin.

Since current therapies are ineffective in preventing degeneration of dopaminergic neurons, it is imperative to identify novel drugs that delay the progression of PD. Phytoconstituents, those of a dietary origin especially present in fruits, vegetables, and spices, have been a subject of intense investigation in recent years for their therapeutic potential in a multitude of age-related chronic ailments such as cardiovascular, endocrine, neoplastic, and neurodegenerative diseases [10–13]. Vanillin (4-hydroxy-3-methoxybenzaldehyde), whose chemical structure is shown in Figure 1, is the principal component of the bean and pod of tropical vanilla orchids (*Vanilla planifolia*,* Vanilla tahitensis*, and* Vanilla pompona*). It is one of the most widely used flavor components in beverage, food preservatives, cosmetics, and drugs industry, with an estimated annual worldwide consumption of more than 2000 tons [14]. Besides its industrial and food application, vanillin exhibits antimicrobial activity [15] as well as antimutagenic [16] and anticarcinogenic actions [17].

Moreover, vanillin can inhibit peroxynitrite-mediated reactions that are known to be involved in the pathogenesis of several neurodegenerative diseases such as Alzheimer's and Parkinson's diseases [18]. Vanillin has been shown to control cognitive decline, mitochondrial dysfunction, oxidative stress, and neurodegeneration in experimental model of Huntington's disease [19]. Vanillin reduces the expressions of proinflammatory cytokines (interleukin-1*β* and interleukin-6, interferon-*β*, and tumor necrosis factor-*α*) and stimulates the expression of anti-inflammatory cytokine (IL-4) in tissues [20]. It has been reported to show significant brain protective property by reducing the levels of reactive oxygen/nitrogen species and augmenting the activities of antioxidant enzymes [21]. The biological properties of vanillin are mainly attributed to the presence of phenolic group, ether and aldehyde moieties. Till now the effect of vanillin on rotenone induced PD like neurotoxicity is not well explored. Moreover* in vitro* rotenone model of PD has been used for identifying potential neuroprotective agents [22] including various herbs [23] and novel antiparkinsonian drugs [24] for treating PD. Based on this, the present study was designed to find out the protective effects of vanillin on the mitochondrial dysfunction, oxidative stress, and apoptotic damage produced in rotenone exposed SH-SY5Y neuroblastoma cells.

## 2. Materials and Methods

### 2.1. Chemicals

Rotenone, vanillin, 3-(4,5-dimethylthiazol-2-yl)-2,5-diphenyltetrazolium bromide (MTT), 2-7-diacetyl dichlorofluorescein (DCFH-DA), rhodamine 123 (Rh-123), heat-inactivated fetal bovine serum (FBS), Dulbecco's modified Eagle's medium (DMEM), antibiotic/antimycotic, EDTA, and Trypsin-EDTA were procured from Sigma Chemicals Co. (St. Louis, USA). Anti-Bcl-2, anti-Bax, caspase-3, caspase-8, caspase-9, cyt-c, and anti-JNK and anti-P38 MAPK antibodies were obtained from Cell Signaling (USA) and *β*-actin, anti-mouse, and anti-rabbit secondary antibodies were purchased from Santa Cruz Biotechnology, Inc. (USA).

### 2.2. Cell Culture

SH-SY5Y cells were obtained from National Center for Cell Science (NCCS), Pune, India. The cells were grown in DMEM supplemented with 10% FBS and 1% antibiotic/antimycotic solution. Cultures were maintained in a humidified incubator at 37°C in an atmosphere of 5% CO_2_ and 95% air. Cell culture medium was changed every 2 days.

### 2.3. Cell Viability Assay

Cell viability assay was determined by MTT assay, as described previously [25]. SH-SY5Y cells were collected and seeded in 96-well plates, at a density of 3 × 10^3^ cells/well. To determine the toxicity of rotenone, cells were incubated with different concentrations of rotenone (5, 10, 50, 100, and 200 nM) and vanillin (5 nM, 10 nM, 20 nM, 50 nM, 100 nM, 200 nM, 500 nM, 1 *μ*M, 10 *μ*M, 100 *μ*M, 200 *μ*M, and 500 *μ*M) for 24 h and MTT assay was performed to detect IC_50_ value of rotenone and vanillin. To assess the therapeutic efficacy of vanillin against rotenone toxicity, cells were pretreated with different concentrations of vanillin (5, 10, 20, 50, 100, and 200 nM) for 2 h and then incubated with rotenone (effective dose) for 24 h. Vanillin was also present during rotenone treatment for additional 24 h. Then all the cells were incubated with MTT final concentration (1 mg/mL of serum-free DMEM medium) at 37°C for 4 h. After the incubation, the medium was removed, and 100 *μ*L of DMSO was added to dissolve the formazan crystals. The absorbance of formazan product was evaluated by spectrophotometer at 570 nm using a microplate reader. Four independent experiments were performed from each group.

Based on the results obtained from cell viability assay, the effective dose of vanillin against rotenone toxicity was utilized to study the effect of vanillin by assessing ROS, MMP, apoptosis, and apoptotic markers protein expression.

### 2.4. Experimental Design (*n* = 4 Experiments)

 Group I: untreated control cells.
 Group II: rotenone (effective dose: 100 nM).
 Group III: vanillin (100 nM) + rotenone (100 nM).
 Group IV: vanillin (100 nM).

### 2.5. Measurement of Intracellular ROS

The levels of endogenous ROS formed in control and experimental cells were estimated by using fluorescence dye (DCFH-DA) [26]. After pretreatment with vanillin (100 nM/mL) for 2 h, the cells (1 × 10^5^ cells/well in 6-well plates) were incubated with rotenone (100 nM/mL) for 24 h and then incubated with 100 *μ*L DCFH-DA for 30 min at 37°C and washed twice with PBS to remove the excess probe; the cells were suspended in glucose-enriched PBS and transferred to a fluoroslide and visualized using a fluorescent microscope. Fluorescent measurements were made with excitation and emission filters set at 485 ± 10 nm and 530 ± 12.5 nm, respectively (Shimadzu RF-5301 PC spectrofluorimeter), and the images were captured using fluorescence microscope [27].

### 2.6. Measurement of Mitochondrial Transmembrane Potential (MMP)

MMP changes were determined by the mitochondrial-specific, incorporation of a cationic fluorescent dye Rh-123 [28]. After treatment with vanillin for 2 h and rotenone for 24 h as previously described, the cells (1 × 10^5^ cells/well in 6-well plates) were changed to fresh medium containing 1 *μ*L of fluorescent dye Rh-123 (5 mmol/L) and kept for 30 min at 37°C. The cells were then collected, washed twice with PBS, and estimated by using blue filter (450–490 nm) (Shimadzu RF-5301 PC spectrofluorimeter).

### 2.7. Apoptosis Analysis Using Dual Staining

Dual staining method is used to analyze the apoptotic morphological changes by treating the control and experimental cells with fluorescent probes acridine orange and ethidium bromide (AO and EB) and using fluorescence microscope [29]. After treatment schedule as described in previous experiments, medium was removed from the plates; cells (1 × 10^5^) were washed with PBS twice and then fixed with 4% paraformaldehyde for 20 min and stained with 100 *μ*g/mL AO and EB. These cells were incubated for 20 min at room temperature and washed with warm PBS to remove excess dye. Cellular morphology was examined using fluorescence microscopy and photographed and quantified at 535 nm in spectrofluorometer.

### 2.8. Immunoblotting and Image Analysis

SH-SY5Y cells (1 × 10^5^) in 6-well plates, after 2 h pretreatment with vanillin and 24 h treatment with rotenone, were harvested, washed with PBS, and lysed in 100 *μ*L lysis buffer (20 mM Tris-HCl, pH 7.4, 150 mM NaCl, 1 mM EDTA, 30 *μ*g/mL apoprotein, and 1 mM phenylmethylsulfonyl fluoride) followed by centrifugation at 1,000 g for 5 min at 4°C. The supernatants (cytosolic fractions) were saved and the pellets solubilized in the same volume of mitochondrial lysis buffer (50 mM Tris pH 7.4, 150 mM NaCl, 2 mM EDTA, 2 mM EGTA, 0.2% Triton X-100, 0.3% NP-40, 100 *μ*M PMSF, 10 *μ*g/mL leupeptin, and 2 *μ*g/mL apoprotein) kept on ice and vertex for 20 min followed by pelleting at 10000 g for 10 min at 4°C and subjected to 12.5% polyacrylamide gel electrophoresis lane [13]. The separated proteins were blotted onto a PVDF membrane by semidry transfer (BIORAD). After blocking with 5% nonfat milk in TBS at 25°C for 1 h, blots were probed with various antibodies: caspase-3, caspase-8, and caspase-9, cytochrome c, Bax, Bcl_2_, p-JNK, and p-P38 and p-ERK (1 : 1,000) and *β*-actin (1 : 2,000). Horseradish peroxidase-conjugated anti-mouse or anti-rabbit IgG were used as the secondary antibodies at a concentration of 1 : 2,000. Then the membranes were washed with Tris-buffered saline and 0.05% Tween-20 thrice for 10 min interval, after extensive washes in TBST. Bands were scanned using a scanner and quantified by Image J, a public domain Java Image processing software, Wayne Rasband, NIH, Bethesda, MD, USA, in which the control group was set to100% [30].

### 2.9. Data Analysis

Statistical analysis was performed by one-way analysis of variance followed by Duncan's multiple range test (DMRT) using Statistical Package for the Social Science (SPSS) software package version 12.0. Results were expressed as mean ± SD for four experiments in each group. *p* < 0.05 were considered significant.

## 3. Results

### 3.1. Cytotoxicity of Rotenone in SH-SY5Y Cells

**Figure 2:**
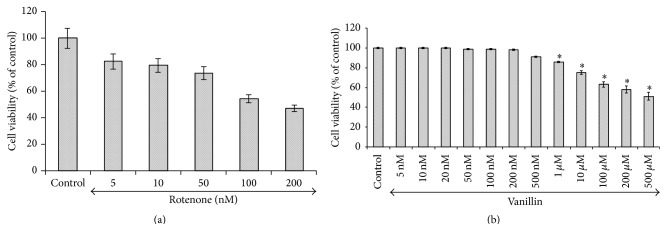
Effect of vanillin on rotenone induced cytotoxicity (MTT assay) in SH-SY5Y neuroblastoma cells. Cell viability was determined by measuring MTT method. (a) shows the dose-dependent effect of rotenone (5, 10, 50, 100, and 200 nM) induced cell toxicity after 24 h. An approximately half-maximal inhibition of cell viability was obtained at 100 nM rotenone concentration. (b) shows the dose-dependent effect of vanillin at various concentrations. Low concentrations (5, 10, 20, 50, 100, and 200 nM) did not induce any toxicity after 24 h treatment, whereas slight toxicity was induced at 500 *μ*M concentration. Values are expressed as the percentage of the untreated control and represented as mean ± SD of four independent experiments in each group.

A dose-dependent cytotoxic effect of rotenone was evaluated by MTT assay in SH-SY5Y human neuroblastoma cells, which measures mitochondrial function or integrity with a dose of 100 nM which caused ~50% of cell death as compared with controls and was taken as inhibitory dose (Figures 2(a) and 2(b)).

### 3.2. Vanillin Protects Rotenone Induced SH-SY5Y Cell Death

**Figure 3:**
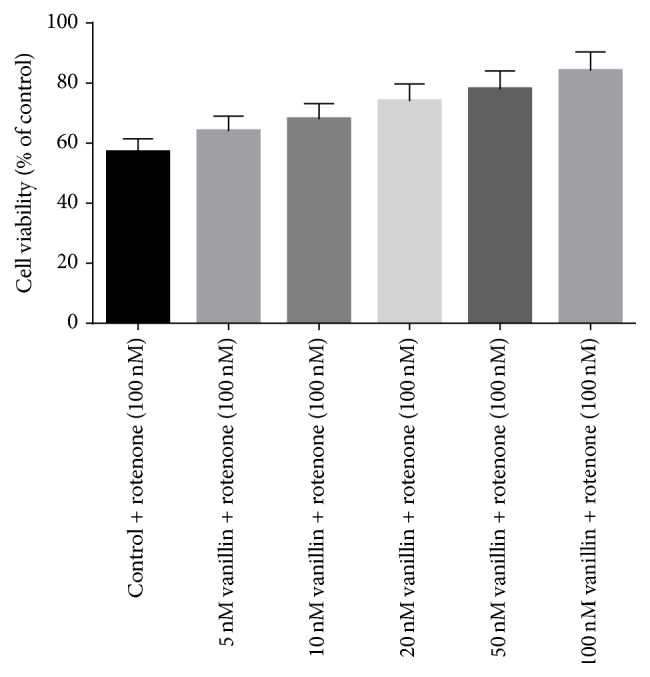
The protective effect of vanillin (5, 10, 20, 50, and 100 nM) against rotenone induced cell death was determined by MTT assay. Values are expressed as the percentage of the untreated control and represented as mean ± SD of four independent experiments in each group.

Figure 3 shows the protective effect of vanillin against rotenone induced injury (100 nM) with cell viability increasing to 84 ± 6.7% of control in the presence of 100 nM vanillin. So based on the dose-response data, the treatments of 100 nM vanillin and 100 nM rotenone were chosen for further experiments (Figure 3).

### 3.3. Vanillin Attenuates Rotenone Induced ROS Generation

**Figure 4:**
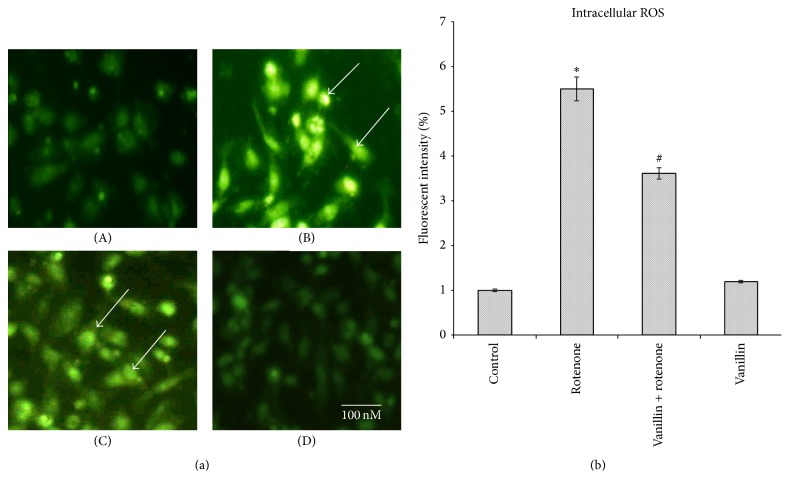
(a) Vanillin reduced ROS formation as stained by 1 *μ*M CM-H2 DCFDA. (a) Photomicrograph showing the preventive effect of vanillin (100 nM) against rotenone induced ROS generation. (A) Control, (B) rotenone, (C) vanillin + rotenone, and (D) vanillin. (b) Rotenone (100 nM) treatment significantly increased the levels of ROS as compared to control cells, while vanillin (100 nM) pretreatment significantly decreased the levels of ROS as compared to rotenone alone treated cells. Values are given as mean ± SD of four independent experiments in each group. ^*∗*^ *p* < 0.05 compared to control and ^#^ *p* < 0.05 compared to rotenone group (DMRT).

To analyze the effect of vanillin on free radical generation, the levels of intracellular ROS formed were quantified by fluorescence with H_2_DCF-DA. Rotenone treatment enhanced the green fluorescence, an indicator of high levels of ROS, and pretreatment of vanillin to rotenone exposed cells revealed reduced green color intensity, an indicator of decreased intracellular ROS formation (Figures 4(a) and 4(b)).

### 3.4. Vanillin Ameliorates Rotenone Induced Mitochondria Membrane Potential

**Figure 5:**
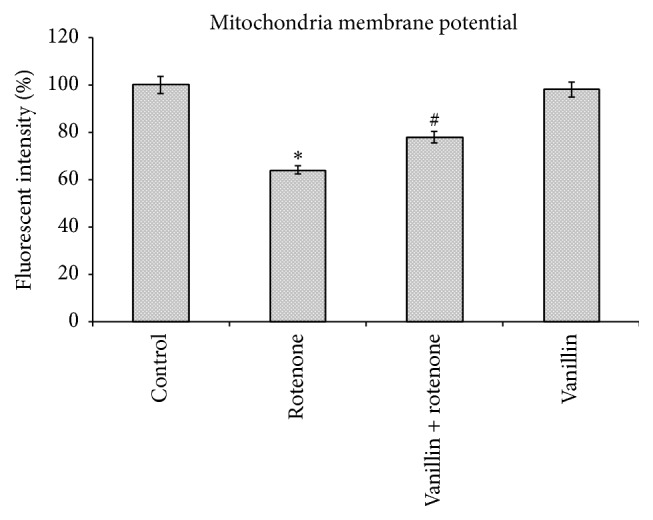
Vanillin stabilizes MMP as stained by Rh-123. Rotenone (100 nM) significantly decreased mitochondria membrane potential, while cells that were pretreated with vanillin (100 nM) significantly increased MMP. Values are given as mean ± SD of four independent experiments in each group. ^*∗*^ *p* < 0.05 compared to control; ^#^ *p* < 0.05 compared to rotenone groups (DMRT).

Alteration in the MMP is considered to be one of the important events related to apoptosis. The effect of vanillin on MMP in rotenone induced toxicity was analyzed by measuring the uptake of Rh-123. In normal cells, Rh-123 steadily penetrates the cells, stains mitochondria, and exhibits high fluorescent intensity. The depolarization of MMP due to rotenone treatment results in the loss of Rh-123 from the mitochondria and a decrease in intracellular green fluorescence. Cell cultures pretreated with vanillin before rotenone treatment partially reduced this decline in fluorescence and approached control levels (Figure 5).

### 3.5. Vanillin Shields Rotenone Induced Apoptotic Changes (Cellular Morphology)

**Figure 6:**
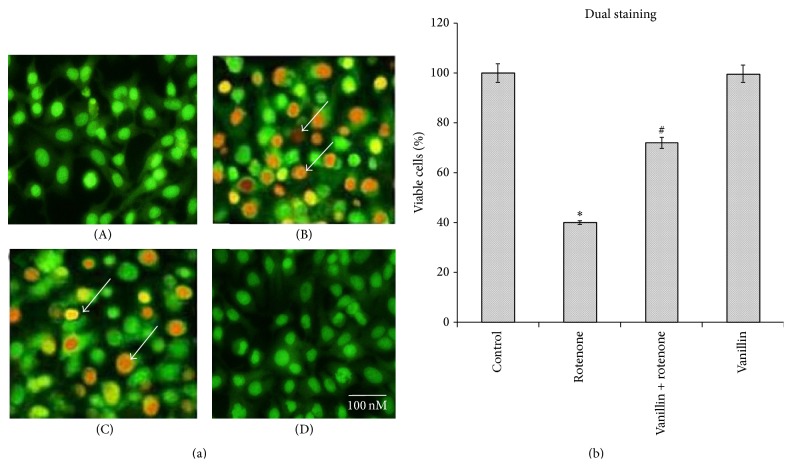
Vanillin protects SH-SY5Y cells against rotenone induced apoptosis. (a) Photomicrograph showing the antiapoptotic effect of vanillin (100 nM) against rotenone at a concentration of 100 nM effective dose. (A) Control, (B) rotenone, (C) vanillin + rotenone, and (D) vanillin. (b) Rotenone (100 nM) treatment induced cell apoptosis compared to control cells; pretreatment with vanillin (100 nM) suppresses these apoptotic features. Values are given as mean ± SD of four independent experiments in each group. ^*∗*^ *p* < 0.05 compared to control and ^#^ *p* < 0.05 compared to rotenone group (DMRT).

Double staining of rotenone and vanillin treated SH-SY5Y cells, with AO and EB, was used to determine the rate of apoptosis. Control cells which fluoresced brightly with green nuclei and normal morphology were showed in Figure 6(a). In contrast, at 100 nM rotenone exposure, cells revealed orange luminescent apoptotic body formation, when compared to control (*p* < 0.05), and treatment with vanillin increased cell viability and decreased apoptotic cell death when compared to cells exposed merely to rotenone (Figure 6(b)).

### 3.6. Vanillin Prevents Rotenone Induced Changes in Protein Expressions of Apoptotic and Signaling Markers

**Figure 7:**
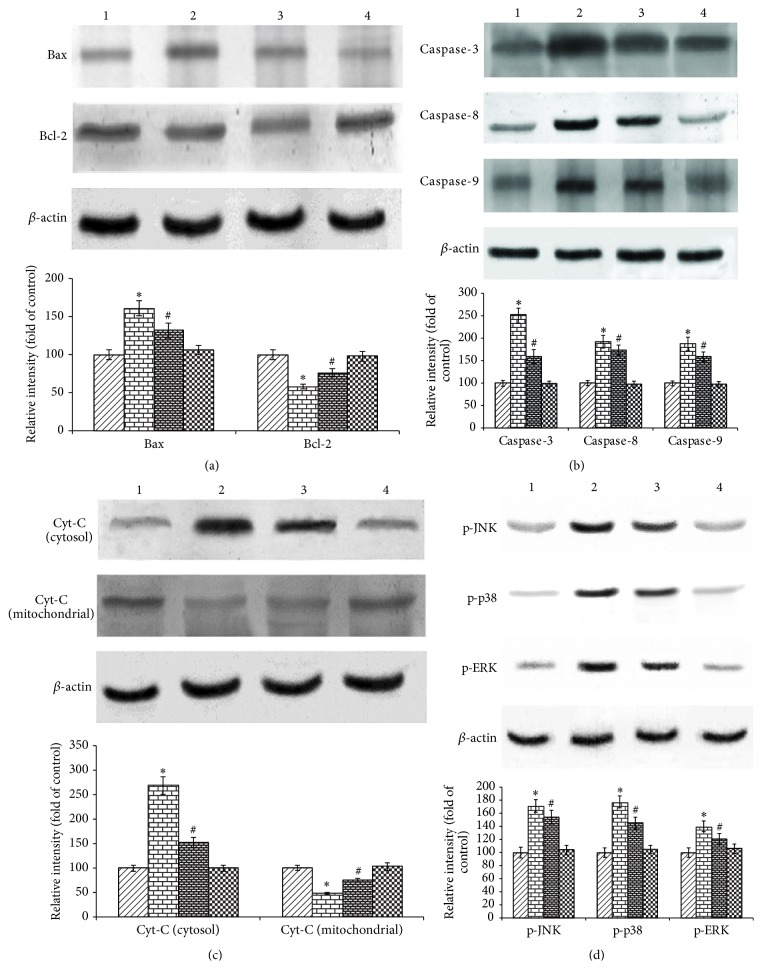
The effect of vanillin on the expressions of apoptotic and signaling markers. Lane 1: control; 2: rotenone; 3: vanillin + rotenone; and 4: vanillin. (a), (b), and (c) show the expressions of Bax; caspase-3, caspase-8, and caspase-9 cyt-c in cytosol were increased while the expressions of Bcl-2 and cyt-c in mitochondria were significantly decreased by the rotenone treated group as compared with control. Pretreatment with vanillin gradually restored the imbalanced expression profile of these proteins. (d) Rotenone treatment stimulates the expressions of p-JNK, p-P38, and p-ERK as compared with control. Pretreatment with vanillin decreases the expressions of p-JNK, p-P38, and p-ERK significantly. Immunoblots are representative of at least four independent experiments. Values are given as mean ± SD in each group. ^*∗*^ *p* < 0.05 compared to control and ^#^ *p* < 0.05 compared to rotenone group (DMRT).

To further characterize the mechanism of inhibition by vanillin on rotenone induced apoptosis, we determined the effect of vanillin on the expression of anti- and proapoptotic proteins by western blot. The expression of Bax, caspase-3, caspase-8, and caspase-9 was increased while the distribution of Bcl-2 and cyt-c in mitochondria was significantly decreased by the rotenone treated group as compared with control. Rotenone treatment significantly diminished the translocation of cyt-c in cytosol. Pretreatment with vanillin gradually restored the imbalanced expression profile of these proteins. Vanillin, which had no effects in control cells, in contrast, when treated with rotenone noticeably, changes the protein expressions in SH-SY5Y cells (Figures 7(a), 7(b), and 7(c)). In order to elucidate the mechanism of rotenone induced cell death and protective effective of vanillin, the protein expression studies of signaling molecules were performed. Expressions of p-JNK, p-P38, and p-ERK were significantly increased after rotenone treatment as compared with control. Following pretreatment with vanillin before rotenone addition is able to decrease the levels of p-JNK, p-P38, and p-ERK significantly. However, treatment with vanillin alone did not alter the expression; p-JNK, p-P38, and p-ERK were unaffected as compared to control (Figure 7(d)).

## 4. Discussion

Results of the present study indicated that the rotenone treatment for 24 h destroyed SH-SY5Y cells in a dose-dependent manner and approximately half-maximal inhibition of cell viability (~54.41%) was obtained at 100 nM rotenone concentration, which corroborated our previous studies [13]. The observed condition mimics the situation at the time of initial diagnosis of PD, when approximately 50% of neurons in the substantia nigra are alive, although many of them may be undergoing subcellular stress [31]. In the present study, 2 h prior exposure of vanillin significantly enhanced cell viability in a dose-dependent manner. In this study, the IC_50_ of vanillin was found at 500 *μ*M. However, Ho et al. [17] and Lirdprapamongkol et al. [32] showed that the IC_50_ value of vanillin is 400 *μ*M, on colorectal cancer cell line HT-29 and breast cancer cell line, which indicated its safety. Kim et al. reported that the treatment of vanillin increased undifferentiated neuronal (PC-12) survival against H_2_O_2_ toxicity [33], which is in line with our studies.

Most of the existing reports have suggested that the mitochondrial dysfunction and oxidative stress play a key role in the pathogenesis of neurodegeneration in PD [34]. The mitochondrial respiratory chain (complexes I and III) is one of the most important sites of ROS production, in which complex I could be a critical site of mitochondrial ROS production and relatively small level of inhibition is sufficient to increase ROS generation [35, 36]. Rotenone is known to bind to complex I of the electron transport chain and prevent the transfer of electrons from iron sulfur clusters to ubiquinone [37], subsequently causing electrons to accumulate within respiratory chain components. These electrons can be added directly to oxygen molecules to produce superoxide (O_2_^−∙^) anion [38]. Formation of (O_2_^−∙^) is also expected to be enhanced significantly via inhibition of NADH dehydrogenase and enhanced activity of NAD(P)H oxidization [39]. Superoxide dismutase (SOD) catalyzes the dismutation of O_2_^∙−^ to H_2_O_2_. Subsequently, H_2_O_2_ is quickly reduced to water by two other enzymes, catalase and glutathione peroxidase (GPx). In the present study, increased levels of ROS observed in the rotenone model indicated that oxidative stress was induced by rotenone and is attenuated by treatment of vanillin which might be because of its free radical scavenging activity. Though vanillin is a potent antioxidant, its alone treatment to SH-SY5Y cells triggers the levels of ROS nonsignificantly. Results of the present study corroborate previous experiments, in which the addition of celastrol, a triterpenoid [40], rutin, a quercetin glycoside [41], and baicalein, a flavonoid [42], alone increased the levels of ROS. Treatment with vanillin ameliorated 3-nitropropionic acid induced impaired mitochondrial enzyme complexes (I, II, and IV) in experimental model of Huntington's disease [19]. Further it could inhibit singlet oxygen-induced protein and lipid oxidation [43, 44]. These studies support our current observations.

Rotenone is reported to induce dopaminergic neuronal apoptosis through activation of mitogen-activated protein kinase (MAPK) pathway and caspase-dependent pathway [45, 46]. Intact MMP is necessary to maintain closure of the multiprotein pore and the mitochondrial permeability transition pore [30, 47]. Loss of MMP could lead to opening of the mitochondrial permeability transition pore, through which the cytochrome c is released into the cytosol. Subsequently it forms apoptosome complexes with Apaf-1, dATP, caspase-3, and caspase-9 [48]. Cyt-c release also activates proenzyme caspase-9 (initiator) which subsequently leads to activation of apoptosis executioner caspase-3. The active caspase-3 promotes apoptosis by endonucleases activation resulting in cleavage of cellular substrates and ultimately neuronal death as seen in PD [29].

In the apoptotic cells, Bax, as a proapoptotic protein, interacts with Bid and resulting conformational changes cause the translocation of Bax to the outer mitochondrial membrane where they oligomerize and form protein-permeable channels. This further promotes cell death by releasing cytochrome c and other lethal factors from the mitochondria [49, 50]. Bcl-2 is an antiapoptotic protein that prevents the loss of MMP, a key event in apoptosis thereby inhibiting the release of cytochrome c and activation of caspases. Caspases (cysteinyl aspartate-specific proteases) are synthesized as enzymatically inactive precursor proteins (procaspase) and after their activation by proteolytic process leading to apoptosis. Moreover, pro-caspase-2, pro-caspase-3, pro-caspase-8, and pro-caspase-9 have been found to be abundant in the mitochondrial intermembrane space depending on the cell type. We observed the expressions of caspase-3, caspase-8, and caspase-9 in the untreated SH-SY5Y neuroblastoma cells, which corroborated previous reports [40, 51]. The characteristic features of apoptosis in rotenone treated SH-SY5Y cells observed in our study are the loss of MMP, enhanced release of cytochrome c, upregulated expressions of caspase-9, caspase-3, and Bax, and diminished expression of Bcl-2. Vanillin pretreatment reversed those apoptotic markers status in our study and showed the possible antiapoptotic properties of vanillin, which may be due to its mitochondrial protective action.

MAPK subfamilies including extracellular-signal regulated kinase 1/2 (ERK1/2), c-Jun NH2-terminal kinase (JNK), and P38 MAPK play diverse roles in the neuronal differentiation, survival, and death [46, 52]. In the present study, treatment with rotenone induces apoptosis by stimulating the phosphorylation of intracellular 38/JNK MAPK proteins. The activation of 38/JNK MAPK proteins, in turn, induces the apoptosis by recruitment of cytoplasmic Bax to mitochondria and forms permeability transition pore, causing the release of cytochrome c (a death messenger in the cytosol), which further stimulates apoptosis [46, 48, 52, 53]. After the entry of Bax into mitochondria, it gets phosphorylated by JNK- and/or p38K-signaling molecules; the relative contents of Bax in mitochondria may be a key marker for mitochondrial membrane permeability (MMP) change and apoptosis [54]. Cheng et al. reported that the gene ontology and network analysis revealed that genes associated with cancer progression were downregulated by vanillin and suggested that vanillin could be able to influence the regulation of cell cycle and apoptosis signaling molecule in human hepatocarcinoma cells [55].

As a potent insecticide, rotenone primarily inhibits cellular respiratory metabolism in insect tissues, by acting on the nerve cells and then on muscles [56]. Rotenone inhibits the respiration of NADH-linked substrata (excepting succinate) on isolated mitochondria and is not primarily concerned with oxidative phosphorylation [57]. Fukami and Tomizawa [58] concluded that the noted inhibition of* in vitro* oxidation of L-glutamate in the mitochondrial fraction of insect muscle by rotenone was due to inhibition of glutamic dehydrogenase. In this study, though the impact of vanillin on rotenone depleted activity of GDH is not studied, Lee et al. reported that the vanillin treatment inhibited both intracellular Ca^2+^ rise and apoptosis induced by glutamate [59]. There were many studies published to show the benefit of natural products and their active components on various neurodegenerative diseases including PD, which further supports the current results [60–66].

Although PD was long considered a nongenetic disorder of “sporadic” origin, 5–10% of patients are now known to have monogenic forms of the disease. At least 13 loci and 9 genes are associated with both autosomal dominant (PARK1 and PARK4/*α*-Synuclein; PARK5/UCHL1; PARK8/LRRK2; PARK11/GIGYF2; PARK13/Omi/Htra2) and autosomal recessive (PARK2/Parkin; PARK6/PINK1; PARK7/DJ-1; PARK9/ATP13A2) PD [67]. To develop the cause-directed therapies, further investigations of the effect of vanillin on expression of these genes are required.

## 5. Conclusion

Beyond its wide usage and commercial values, our findings suggested that vanillin could counteract apoptosis induced by rotenone by preserving mitochondrial functions and antioxidant action. Though rotenone induced* in vitro* model of PD is considered as a good model, which resembles various pathological features of clinical studies, further extensive research is warranted to find out the effect of vanillin in animal and clinical models of PD.
